# Altered PPARγ Expression Promotes Myelin-Induced Foam Cell Formation in Macrophages in Multiple Sclerosis

**DOI:** 10.3390/ijms21239329

**Published:** 2020-12-07

**Authors:** Elien Wouters, Elien Grajchen, Winde Jorissen, Tess Dierckx, Suzan Wetzels, Melanie Loix, Marie Paule Tulleners, Bart Staels, Piet Stinissen, Mansour Haidar, Jeroen F.J. Bogie, Jerome J.A. Hendriks

**Affiliations:** 1 Department of Immunology and Infection, Biomedical Research Institute, Hasselt University, 3590 Diepenbeek, Belgium; elien.wouters@uhasselt.be (E.W.); elien.grajchen@uhasselt.be (E.G.); winde.jorissen@gmail.com (W.J.); tess.dierckx@uhasselt.be (T.D.); melanie.loix@uhasselt.be (M.L.); mariepaule.tulleners@uhasselt.be (M.P.T.); piet.stinissen@uhasselt.be (P.S.); mansour.haidar@uhasselt.be (M.H.); Jeroen.Bogie@uhasselt.be (J.F.J.B.); 2 Department of Pathology, CARIM, Maastricht University Medical Center, 6229 HX Maastricht, The Netherlands; suzan.wetzels@gmail.com; 3 University of Lille, Inserm, CHU Lille, Institut Pasteur de Lille, U1011-EGID, F-59000 Lille, France; bart.Staels@pasteur-lille.fr

**Keywords:** multiple sclerosis, myelin-loaded macrophages, inflammation, PPARγ

## Abstract

Macrophages play a crucial role during the pathogenesis of multiple sclerosis (MS), a neuroinflammatory autoimmune disorder of the central nervous system. Important regulators of the metabolic and inflammatory phenotype of macrophages are liver X receptors (LXRs) and peroxisome proliferator-activated receptors (PPARs). Previously, it has been reported that PPARγ expression is decreased in peripheral blood mononuclear cells of MS patients. The goal of the present study was to determine to what extent *PPARγ*, as well as the closely related nuclear receptors *PPARα* and *β* and *LXRα* and *β*, are differentially expressed in monocytes from MS patients and how this change in expression affects the function of monocyte-derived macrophages. We demonstrate that monocytes of relapsing-remitting MS patients display a marked decrease in *PPARγ* expression, while the expression of *PPARα* and *LXRα/β* is not altered. Interestingly, exposure of monocyte-derived macrophages from healthy donors to MS-associated proinflammatory cytokines mimicked this reduction in *PPARγ* expression. While a reduced *PPARγ* expression did not affect the inflammatory and phagocytic properties of myelin-loaded macrophages, it did impact myelin processing by increasing the intracellular cholesterol load of myelin-phagocytosing macrophages. Collectively, our findings indicate that an inflammation-induced reduction in *PPARγ* expression promotes myelin-induced foam cell formation in macrophages in MS.

## 1. Introduction

Multiple sclerosis (MS) is a neurodegenerative autoimmune disease of the central nervous system (CNS) characterized by chronic inflammation, demyelination, and axonal degeneration. During MS, circulating monocytes are recruited to the site of CNS inflammation. At the lesion site, monocyte-derived macrophages (MDMs) mediate demyelination and neurodegeneration [1]. Conversely, MDMs can also stimulate repair by internalizing myelin debris or by producing anti-inflammatory and repair-promoting factors [2,3,4,5]. After the internalization of myelin, cholesterol—the main lipid constituent of myelin—is transported out of the cell via ATP-binding cassette transporters (e.g., ABCA1 and ABCG1) [6]. The efflux of cholesterol is essential to maintain intracellular lipid homeostasis and to prevent MDMs from becoming foamy. Nevertheless, a vast number of foamy macrophages containing abundant myelin remnants are found within demyelinating plaques of MS patients, indicating that lipid homeostasis is dysregulated in these cells [5,7].

Previously, we found that myelin uptake by bone marrow-derived mouse macrophages results in the activation of lipid sensing nuclear receptors (NRs), called peroxisome proliferator-activated receptors (PPARs) [8]. PPARs are ligand-activated transcription factors that control both lipid metabolism and inflammation [9]. Three PPAR isoforms have been identified, namely, PPARα (NR1C1), PPARβ/δ (NR1C2), and PPARγ (NR1C3). Upon ligand binding, PPARs form heterodimers with retinoic X receptors (RXRs) and bind to specific DNA regions, called hormone response elements, resulting in the transcriptional regulation of target genes. PPAR activation in macrophages results in the active transcription of genes involved in lipid uptake, transport, synthesis, and efflux, thereby controlling cellular lipid homeostasis [10]. Additionally, PPAR activation inhibits proinflammatory signaling pathways [11].

It is previously reported that PPARγ protein level is decreased in peripheral blood mononuclear cells (PBMCs) of MS patients [12]. Furthermore, stimulation of PBMCs with phytohemagglutinin (PHA), a mitogen that stimulates inflammation, significantly reduces PPARγ protein abundance [12]. In this study, we demonstrate that the expression of *PPARγ* is decreased in monocytes of relapsing-remitting MS (RR-MS) patients and that MS-associated proinflammatory cytokines IFNγ and IL1β reduce *PPARγ* expression. In addition, we find that PPARγ deficiency in myelin-containing macrophages has a significant impact on myelin processing by increasing the intracellular cholesterol load in these cells. Our findings highlight the importance of PPARγ in maintaining the intracellular lipid homeostasis in macrophages.

## 2. Results

### 2.1. The Inflammatory Environment in MS Reduces Macrophage PPARγ Expression

**Figure 1:**
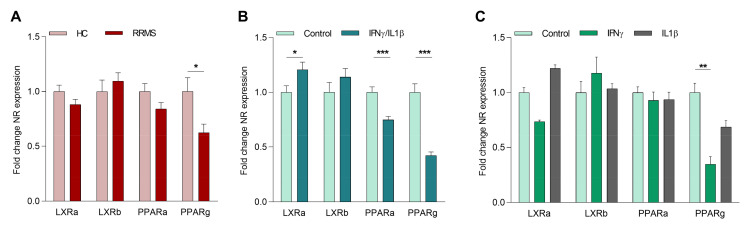
Inflammatory cytokines decrease *PPARγ* expression in macrophages. (**A**) Basal mRNA expression of *LXR-* and *PPAR*-isoforms in monocytes from age- and gender-matched healthy controls and relapsing-remitting multiple sclerosis (MS) patients (*n* = 15). (**B**) Gene expression of *LXR*- and *PPAR*-isoforms in monocyte-derived macrophages (MDMs) from healthy controls stimulated with IFNγ/IL1β (*n* = 11). (**C**) Gene expression of *LXR*- and *PPAR*-isoforms in MDMs from healthy controls stimulated with IFNγ or IL1β compared to nonstimulated cells (*n* = 4). RR-MS = relapsing-remitting MS patient; NR = nuclear receptor. Values represent the mean ± S.E.M. Statistical significance (**A**,**B**; unpaired Student’s *t*-test, and **C**; one-way ANOVA with Dunn’s multiple comparison correction) is indicated with asterisks: * *p* ≤ 0.05, ** *p* ≤ 0.01, and *** *p* ≤ 0.001. *n* represents the total number of donors included in the experiment.

We determined to what extent *PPARγ*, as well as the closely related nuclear receptors *PPARα, β* and *LXRα, β*, are differentially expressed in monocytes from MS patients. For this purpose, CD14 positive cells from age- and gender-matched healthy donors and MS patients were isolated. Our data show that the mRNA expression of *PPARγ*, but not *LXRα*, *LXRβ*, nor *PPARα* was lower in monocytes of RR-MS patients compared to healthy donors (Figure 1A). No correlation of *PPARγ* expression with gender, disease activity, or therapy was observed in our samples. A negative correlation for *LXRβ* and age was found (Supplementary Figure S1). However, given the relatively small sample size, future studies should confirm the latter finding using a larger study cohort and identify possible correlations with other factors. No detectable levels of *PPARβ* were found in control and MS patient-derived monocytes (undetermined Ct values). Interestingly, monocytes from secondary progressive (SP)-MS patients did not display a reduced expression of *PPARγ* (Supplementary Figure S2A). Furthermore, the reduced *PPARγ* expression in monocytes of RR-MS patients was lost upon differentiation into macrophages in vitro *(*Supplementary Figure S2B). These findings suggest that disease-associated factors present in RR-MS patients, but not in SP-MS patients and in vitro cultures, maintain reduced *PPARγ* expression in myeloid cells.

Given overt systemic inflammation in RR-MS patients compared to SP-MS patients, we next defined whether inflammation impacts the expression of the different *LXR*- and *PPAR*-isoforms. Our data show that the expression of *PPARγ* as well as *PPARα* is suppressed by the inflammatory cytokines IFNγ and IL1β in in vitro differentiated MDMs. In contrast, the expression of *LXRs* was not influenced by inflammatory cytokines (Figure 1B). To investigate the individual effect of both cytokines, cells were treated with either IFNγ or IL1β. Here, we observed that IFNγ most potently decreased *PPARγ* expression (Figure 1C). Taken together, these results strongly suggest that inflammation reduces the expression of *PPARα* and *PPARγ* in myeloid cells of MS patients, and that *PPARγ* is most susceptible to the inhibitory impact of inflammatory mediators.

### 2.2. Myelin Uptake Activates PPARγ in Phagocytes

**Figure 2:**
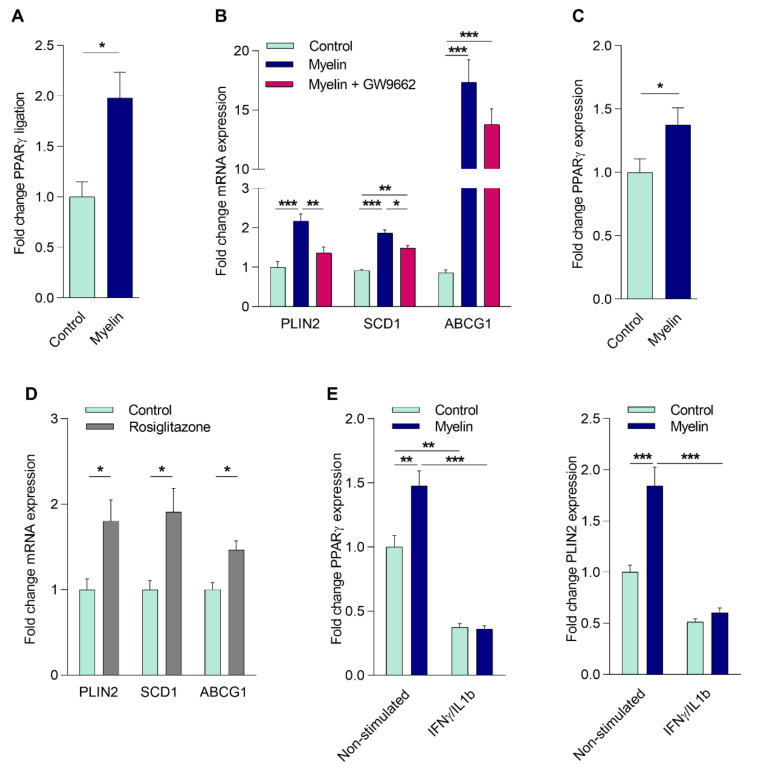
Myelin uptake activates PPARγ in phagocytes. (**A**) CHME3 cells treated with myelin or left untreated. PPARγ ligand-binding activity was determined using the GAL-4-PPARγ chimera assay system (*n* = 5). (**B**) mRNA expression of PPARγ response genes in MDMs from healthy controls (HCs) stimulated with myelin in the presence or absence of GW9662 (25 µM; *n* = 8). (**C**) *PPARγ* mRNA expression in MDMs from HCs treated with myelin compared to non-stimulated cells (*n* = 11). (**D**) mRNA expression of PPARγ response genes in MDMs from HCs stimulated with rosiglitazone (1 µM; *n* = 9). (**E**) *PPARγ* and perilipin 2 (*PLIN2*) expression in MDMs from HCs treated with IFNγ/IL1β, followed with or without stimulation with myelin (*n* = 6). Values represent the mean ± S.E.M. Statistical significance (**A**,**C**,**D**; Mann–Whitney test, **B**; one-way ANOVA with Tukey’s multiple comparison correction, and **E**; two-way ANOVA with Sidak’s multiple comparison correction) is indicated with asterisks: * *p* ≤ 0.05, ** *p* ≤ 0.01, and *** *p* ≤ 0.001. *n* represents the total number of biological replicates (**A**) or donors (**B**–**E**) included in the experiment.

In the CNS of MS patients, infiltrated monocytes differentiate into macrophages and actively phagocytose myelin [5]. Given that fatty acids are present in myelin and are well-known endogenous ligands of PPARs, we next sought to determine if altered PPARγ activity affects the physiology of myelin-phagocytosing macrophages. For this purpose, we first determined whether myelin internalization activates PPARγ in these cells. To this end, MDMs from healthy donors (HCs) were exposed to isolated human myelin and/or the PPARγ specific agonist (rosiglitazone) and antagonist (GW9662). First, PPARγ activation through its ligand-binding domain was studied using a GAL-4-PPARγ LBD chimera reporter assay in cells treated with myelin for 24 h. As depicted in Figure 2A, myelin treatment resulted in a 2-fold increase in PPARγ activity. Given that in vitro differentiation into macrophages abolishes the reduced *PPARγ* expression observed in monocytes of RR-MS patients (Supplementary Figure S2B), and the fact that monocytes are notoriously inefficient phagocytes, we were unable to study the direct impact of reduced PPAR signaling in MS-derived monocytes on their function. Therefore, we studied if the PPAR antagonist GW9662 altered the macrophage response to myelin. Myelin induced the expression of the PPAR responsive genes *perilipin 2* (*PLIN2*) and *stearoyl-CoA desaturase-1* (*SCD1*). This induction was reduced by GW9662 (Figure 2B). While *ABCG1* expression was also increased upon myelin internalization, this did not depend on PPARγ activation (Figure 2B). Of interest, myelin also increased the expression of *PPARγ* (Figure 2C), suggesting a positive feedback loop that augments PPARγ signaling. The expression of *PLIN2*, *SCD1,* and *ABCG1* was also increased upon rosiglitazone treatment, confirming that these genes are regulated through PPARγ (Figure 2D). Next, we defined if an inflammatory environment and the consequent reduction in *PPARγ* expression impacts the capacity of myelin to induce *PPARγ* and the PPARγ-responsive gene *PLIN2* in MDMs from HCs. Our data demonstrate that myelin does not significantly induce *PPARγ* and *PLIN2* expression under inflammatory conditions (Figure 2E). Collectively, these data illustrate that myelin internalization results in the activation of PPARγ, and that the inflammation-associated reduction in *PPARγ* expression reduces the capacity of myelin to (auto)regulate *PPARγ* and *PLIN2* expression. 

### 2.3. Myelin Controls the Inflammatory Phenotype of Macrophages Independently of PPARγ

**Figure 3:**
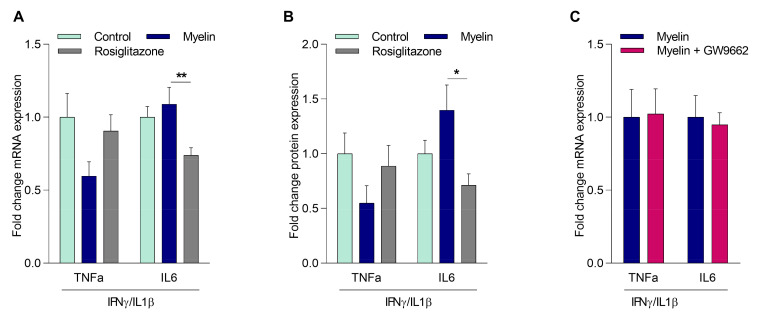
Myelin controls the inflammatory phenotype of mye-macrophages independently of PPARγ. MDMs from HCs treated with myelin, rosiglitazone, or left untreated, followed by incubation with IFNγ/IL1β (*n* = 6). *TNFα* and *IL6* mRNA expression levels (**A**) and protein expression levels (**B**) were detected. (**C**) *TNFα* expression in MDMs from HCs treated with myelin in the presence or absence of GW9662, followed by incubation with IFNγ/IL1β (*n* = 4). Values represent the mean ± S.E.M. Statistical significance (**A**,**B**; one-way ANOVA with Dunn’s multiple comparison correction, and **C**; Mann–Whitney test) is indicated with asterisks: * *p* ≤ 0.05, and ** *p* ≤ 0.01. *n* represents the total number of donors included in the experiment.

Ample evidence indicates that myelin internalization modulates inflammatory gene expression in macrophages [8,13]. As PPARγ is well-known to suppress the inflammatory status of macrophages [11], we next defined whether PPARγ activation impacts the inflammatory phenotype of myelin-containing macrophages (mye-macrophages) [11]. Our data show that myelin uptake decreased TNFα expression, both on mRNA and protein level, in MDMs from healthy donors. However, this decrease did not reach statistical significance. In contrast, the PPARγ agonist rosiglitazone reduced IL6 expression (mRNA and protein), without affecting TNFα expression (Figure 3A,B). These results suggest that PPARγ is not the main regulator of myelin-induced changes in inflammatory gene expression. To confirm that myelin impacts the inflammatory phenotype in a PPARγ-independent manner, myelin-treated MDMs from HCs were exposed to the PPARγ antagonist GW9662. Inhibiting PPARγ activity did not change the expression of *TNFα* and *IL6* in myelin-treated MDMs, providing further evidence for the redundant role of PPARγ in driving the expression of these inflammatory genes in mye-macrophages (Figure 3C). Taken together, these data demonstrate that myelin uptake does not change the inflammatory phenotype of macrophages through the activation of PPARγ.

### 2.4. Reduced PPARγ Activity Does Not Impact Myelin Phagocytosis but Hampers Intracellular Lipid Processing

**Figure 4:**
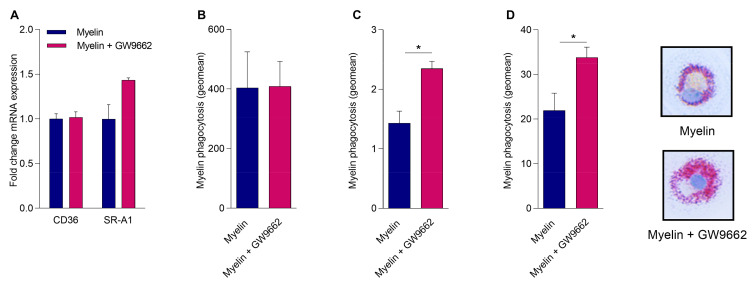
PPARγ controls the formation of lipid droplets in mye-macrophages but does not impact myelin phagocytosis. (**A**) cluster of differentiation 36 (*CD36*) and scavenger receptor-A1 (*SR-A1*) expression in MDMs from HCs pretreated with GW9662 or DMSO, followed by an incubation with myelin (*n* = 5). (**B**) DiI myelin phagocytosis by MDMs from HCs preincubated with GW9662 or DMSO (*n* = 4). (**C**) MDMs from HCs incubated with myelin in the absence or presence of GW9662. Cholesteryl esters (CE) load was measured using the Amplex^TM^ Red Cholesterol Assay (*n* = 4). (**D**) MDMs from HCs incubated with myelin in the absence or presence of GW9662. Neutral lipids were stained and quantified using Oil red O (ORO) (*n* = 5). Values represent the mean ± S.E.M. Statistical significance (Mann–Whitney test) is indicated with asterisks: * *p* ≤ 0.05. *n* represents the total number of donors included in the experiment.

MDMs of MS patients have a reduced capacity to phagocytose myelin [14]. As PPARs control the expression of phagocytic receptors involved in the uptake of myelin such as cluster of differentiation 36 (CD36) and scavenger receptor-A1 (SR-A1) [15,16], we assessed whether PPARγ controls myelin phagocytosis. First, we examined whether myelin-induced PPARγ activation increases the expression of *CD36* or *SR-A1*. By treating MDMs from healthy donors with myelin in the absence or presence of the PPARγ antagonist, we show that PPARγ did not significantly influence *CD36* or *SR-A1* mRNA expression in MDMs upon myelin uptake (Figure 4A). Next, the effect of PPARγ deficiency on the phagocytosis of myelin was determined. For this purpose, MDMs from HCs were exposed to DiI-labeled myelin in the presence or absence of GW9662. No change in DiI myelin phagocytosis was observed when PPARγ was inhibited (Figure 4B). These findings indicate that PPARγ does not regulate myelin internalization. A vast number of foamy macrophages containing abundant myelin remnants are found within demyelinating plaques of MS patients. Since PPARγ has been reported to be a crucial mediator of lipid processing [17,18], we next determined the effect of a reduced PPARγ activity on lipid storage after myelin uptake. By means of the Amplex^TM^ Red Cholesterol Assay, more cholesteryl esters (CEs) were detected inside mye-macrophages from HCs when PPARγ was inhibited (Figure 4C). In line with these results, an increased amount of lipid droplets (LDs) was observed in cells treated with the PPARγ antagonist after myelin uptake (Figure 4D). Similar as before, inefficient phagocytosis by monocytes and the negating effect of monocyte differentiation into macrophages on PPARγ expression did not allow us to confirm these findings directly in MS patient-derived monocytes and macrophages. Collectively, these data indicate that a reduced PPARγ activity promotes the formation of foamy macrophages without affecting the uptake of myelin. 

## 3. Discussion

The activation of NRs such as LXRs and PPARs suppresses neuroinflammation by changing the metabolic and inflammatory properties of immune cells. Here, we show that an inflammation-induced reduction in *PPARγ* expression promotes myelin-induced foam cell formation in macrophages, which may negatively impact MS lesion progression.

Diverse studies demonstrated that inflammatory cytokine levels in biological fluids correlate with disease activity and the occurrence of relapses in MS patients [19,20]. Interestingly, our findings show that MS-associated inflammatory mediators reduce the expression of *PPARγ*, mimicking the *PPARγ* expression profile of monocytes in RR-MS patients. More specifically, IFNγ most prominently reduced *PPARγ* expression. IFNγ is secreted by activated immune cells, including Th1-cells, and is highly elevated in the serum and cerebrospinal fluid (CSF) of MS patients, as well as inside MS lesions [21,22]. Genetic ablation of IFNγ aggravates the disease course in experimental autoimmune encephalomyelitis (EAE), a well-established animal model of MS [23]. However, when IFNγ administration was tested as a clinical drug in MS patients, it resulted in an exacerbation of the disease activity [24,25]. Considering the protective impact of PPARγ activation on neuroinflammation, it is tempting to speculate that IFNγ partially promotes disease severity by reducing monocytic *PPARγ* expression. Our findings are in line with a previous study that showed that PPARγ expression is decreased in PBMCs of MS patients [12]. Collectively, our data show that the proinflammatory environment that is typically associated with MS decreases *PPARγ*, thereby potentially promoting MS disease progression.

PPARγ agonists are well-known to ameliorate disease activity in mice with EAE. In these animals, PPARγ activation reduces CNS inflammation by decreasing the expression of proinflammatory cytokines, including TNFα and IL6 [26,27,28]. In macrophages, PPARγ activation results in the retention of the NCoR corepressor complex at the promotor region of NF-kB target genes, keeping the expression of inflammatory genes in a repressed state [11]. Here, we demonstrate that myelin internalization increases the transcriptional activity of PPARγ in human macrophages. Nonetheless, phenotypic analysis showed that myelin impacts the inflammatory features of mye-macrophages in a PPARγ-independent manner. Based on these findings, the activation of other NRs likely explains the observed effects of myelin on the inflammatory phenotype of macrophages in our study. With respect to the latter, in a previous study we reported that myelin suppresses the inflammatory properties of myelin-containing mouse macrophages in a PPARβ/δ- and LXR-dependent manner [6,8,29]. Collectively, these findings indicate that myelin activates PPARγ but that this activation does not impact the inflammatory phenotype of human macrophages.

Inefficient clearance of myelin debris inhibits oligodendrocyte precursor cell differentiation. Hence, adequate uptake of myelin debris is essential for remyelination to occur [3,30]. Macrophages phagocytose myelin via scavenger receptors including CD36 and SR-A1 [16,31]. Although CD36 and SR-A1 expression is transcriptionally regulated by PPARγ [15,16], we show that myelin uptake does not increase *CD36* or *SR-A1* expression in macrophages, nor did PPARγ inhibition reduce the phagocytosis of myelin. In contrast to our findings, a study by Natrajan et al. demonstrated that PPARγ activation increases the uptake of myelin [32]. However, in the latter study the authors made use of synthetic PPARγ agonists to show that PPARγ activation has an additive effect on myelin phagocytosis. Here, we utilized a PPARγ antagonist to define the role of endogenous PPARγ activity in myelin uptake. Thus, our findings indicate that the reduced myeloid cell expression of PPARγ in RR-MS patients probably does not impact the capacity of these cells to internalize myelin debris.

Alongside controlling the phagocytic and inflammatory features of macrophages, PPARγ mediates the intracellular processing and efflux of lipids. In macrophages present in atherosclerotic plaques, PPARγ activation results in an increase in cholesterol efflux, and ultimately a reduction in foam cell formation [33]. The presence of foamy macrophages is also a characteristic of active MS lesions, indicating dysregulation of lipid metabolism inside these cells. Interestingly, we recently found that sustained intracellular accumulation of myelin debris in macrophages induces a highly inflammatory transcriptional profile [13]. Here, we demonstrate that mye-macrophages display a marked increase in LDs when PPARγ activity is reduced. This increase in LDs could be the result of a decrease in lipid processing or efflux [18]. At the level of lipid processing, PPAR-stimulation induces mitochondrial β-oxidation of fatty acids (FAs) via increasing the expression of carnitine palmitoyl transferase (CPT)1 [34]. A reduction in mitochondrial β-oxidation increases the availability of FAs for neutral lipid synthesis, thereby augmenting lipid droplet formation [35]. Additionally, PPAR activation induces reverse cholesterol transport by increasing the expression of ABCA1, ABCG1, and SR-B1 [18,36,37]. Blocking this efflux will result in the buildup of free cholesterol inside the cell which will be esterified and stored inside LDs to protect the cell from lipotoxicity. However, we did not find a difference in *ABCA1* and *ABCG1* mRNA expression after blocking PPARγ activity. Nevertheless, further research is necessary to study the expression and localization of these transporters at the protein level. 

While our findings link decreased *PPARγ* expression to inflammation, levels of PPARγ and PLIN2 have been reported to be elevated in the spinal cord of EAE mice and in the CSF of MS patients [38,39]. This induction in PPARγ expression might reflect an endogenous protective mechanism to counteract the proinflammatory response during MS or an increase in infiltrating macrophages that express high levels of PPARγ. We show that the expression of LD-associated protein *PLIN2* is increased after PPARγ stimulation. PLIN2 is important for the stability of LDs in macrophages. Nevertheless, mye-macrophages display a marked increase in LDs when PPARγ activity is blocked. These findings indicate that either other factors in the lesion environment impact *PLIN2* expression, or that PPARs also control other (unidentified) processes that contribute to LD processing and biogenesis (e.g., β-oxidation and cholesterol efflux). Therefore, more research is warranted to unravel the molecular mechanisms that underlie the impact of PPARγ on the accumulation of LDs in mye-macrophages.

We found that differentiation of monocytes into macrophages in vitro nullifies the reduced *PPARγ* expression observed in monocytes of RR-MS patients. This finding confirms that the inflammatory microenvironment in RR-MS is essential for maintaining reduced PPARγ activation. This indicates that when inflammatory mediator production is resolved in MS lesions, PPAR function in macrophages will be restored. In future studies, it would be of interest to confirm these findings in post-mortem inflammatory and noninflammatory lesions of MS patients, or compare foamy phagocyte physiology in MS patients to individuals affected by non- or less-inflammatory demyelinating disorders such as adrenoleukodystrophy. 

In conclusion, we report that PPARγ deficiency in mye-macrophages results in an increased intracellular lipid load. This study brings us closer to understanding the mechanisms by which PPARγ can modulate macrophage function and MS lesion progression, and may help in the development of therapeutic strategies to target MS.

## 4. Materials and Methods 

### 4.1. Human Subjects

This study was approved by the Medical Ethics Committees of Hasselt University and UZ Leuven (UH-VAISMS-P1/S56441; March 2014). Written informed consent was obtained from all donors. A total of 22 HCs, 15 RR-MS patients, and 7 SP-MS patients were included in the study (Supplementary Table S1). RR-MS patients participating in the study were in remission at the time of sampling. MS patients were included independent of their treatment. Exclusion criteria for HCs and patients were reported hypercholesterolemia, cardiovascular diseases, diabetes, pregnancy, cancer, liver disease, and treatment with cholesterol modifying agents. 

### 4.2. Cell Culture

PBMCs were isolated from whole blood using density gradient centrifugation, as previously described [40]. CD14^+^ monocytes were collected by positive selection according to the manufacturer’s guidelines (Stemcell Technologies, Grenoble, France). MDMs were obtained by culturing isolated monocytes (2 × 10^6^ cells/mL) in MDM culture medium (DMEM-high glucose medium (Sigma-Aldrich, Diegem, Belgium) supplemented with 10% human serum (Sigma-Aldrich), 50 U/mL penicillin, 50 U/mL streptomycin, and 2 mM L-glutamine (Sigma-Aldrich)) at 37 °C, 5% CO_2_. After 7 days, MDMs were collected using PBS/EDTA (10 mM) and plated at 0.5 × 10^6^ cells/mL in MDM culture medium. 

### 4.3. Human Myelin Isolation

Myelin was isolated from post-mortem nondemented human brain using sucrose density-gradient centrifugation, as previously described [41]. Briefly, tissue was homogenized in 0.32 M sucrose and layered over 0.85 M sucrose. The homogenate was ultra-centrifuged (75,000× *g*, 4 °C, 30 min) and crude myelin was collected from the interface. The collected myelin was washed in ddH_2_O and subjected to osmotic disintegration. A second sucrose density-gradient centrifugation was carried out by dissolving the pellet in 0.32 M sucrose and layering it on top of 0.85 M sucrose. After ultracentrifugation (75,000× *g*, 4 °C, 30 min), the myelin-containing interface was collected, washed in ddH_2_O, and dissolved in PBS. Myelin protein concentration was measured using the BCA protein assay kit (Thermo Fisher Scientific, Erembodegem, Belgium). The amount of endotoxin was determined using the Chromogenic Limulus Amebocyte Lysate assay kit (Genscript Incorperation, Aachen, Germany). Isolated myelin contained a negligible amount of endotoxin (≤1.8 × 10^−3^ pg/µg myelin) [42].

### 4.4. Macrophage Stimulation and Pharmacological Treatments

The effect of a proinflammatory environment on mRNA expression was measured by treating cells with IFNγ and/or IL1β (100 ng/mL, PeproTech, London, UK) for 6 h. To study PPARγ expression and activity, MDMs were stimulated with myelin (100 µg/mL), rosiglitazone (PPARγ agonist, 1 µM, Sigma-Aldrich), or dimethylsulfoxide (DMSO; Leuven, Belgium) for 24 h, in the presence or absence of the PPARγ antagonist, GW9662 (25 µM, Sigma-Aldrich) or IFNγ/IL1β (100 ng/mL). Inflammatory gene expression was measured after treating cells with myelin (100 µg/mL) or rosiglitazone (1 µM) for 18 h, followed by incubation with IFNγ/IL1β (100 ng/mL) for 6 h. GW9662 (25 µM) was added to the myelin conditions to study the involvement of PPARγ in myelin-induced suppression of inflammatory gene expression. 

### 4.5. RNA Extraction and Real-Time Quantitative PCR (RT-qPCR)

Total RNA was isolated with Qiazol (Qiagen, Venlo, The Netherlands) and the RNeasy mini kit (Qiagen), according to the manufacturer’s guidelines. A NanoDrop spectrophotometer (Isogen Life Science, De Meern, The Netherlands) was used to determine RNA concentration and purity. RNA was reverse-transcribed using qScriptTM cDNA SuperMix (Quanta Biosciences, VWR, Leuven, Belgium) according to the manufacturer’s instructions. RT-qPCR was conducted on a Step One Plus detection system (Applied biosystems, Foster City, CA, USA). Cycle conditions were 95 °C for 20 s, followed by 40 cycles of 95 °C for 3 s, and 60 °C for 30 s. The PCR reaction mixture contained SYBR green master mix (Thermo Fisher Scientific), 0.3 μM forward and reverse primer (IDT technologies, Leuven, Belgium), RNase free water, and 12.5 ng cDNA template in a total reaction volume of 10 μL. Relative quantitation of gene expression was accomplished using the comparative Ct method. Data were normalized to the most stable reference genes. Primers used for RT-qPCR are shown in Supplementary Table S2.

### 4.6. Enzyme-Linked Immunosorbent Assay (ELISA)

MDMs were pretreated for 18 h with myelin (100 µg/mL), rosiglitazone (1 µM), or DMSO, followed by an incubation with IFNγ/IL1β (100 ng/mL) for 24 h. Afterwards, medium was collected, centrifuged at 1400 rpm for 10 min, and diluted 1/10. TNFα and IL6 protein expression was measured using ELISA (eBioscience, San Diego, CA, USA). Absorbance was determined by a microplate reader (BIORAD iMarkTM Microplate Reader, Temse, Belgium) at 450 nm. 

### 4.7. Luciferase Assay

Human CHME3 microglial cells were cultured in DMEM-high glucose medium supplemented with 10% fetal bovine serum (Invitrogen, Merelbeke, Belgium), 50 U/mL penicillin, and 50 U/mL streptomycin at 37 °C, 5% CO_2_. CHME3 cells were transfected overnight with plasmids using JetPEI^®^ transfection reagent (Polyplus, Illkirch, France), according to the manufacturer’s protocol. Plasmids used for transfection encoded for pG5-TK-GL3 (GAL5), pCMV-Bgal, and PPARγ (kindly provided by Prof. dr. Bart Staels, University of Lille, France). Following transfection, cells were serum starved for 5 h. Afterwards, CHME3 cells were treated with myelin (100 µg/mL, 24 h) and lysed in lysis buffer (25 mM Gly-Gly (Sigma-Aldrich), 1% Triton (Sigma-Aldrich), 15 mM MgSO4 (Sigma-Aldrich), and 4 mM EGTA (Sigma-Aldrich)). Ligation of myelin components to PPARγ was measured using the ONE-Glo^TM^ Luciferase Assay System kit (Promega, Leiden, The Netherlands). Luminescence was determined with the FLUOstar optima microplate reader (BMG Labtech, Ortenberg, Germany). To study transfection efficacy, β-galactosidase was measured using B-gal buffer (80% Buffer Z (100 mM Na_2_HPO_4_, 10 mM KCl, 1 mM MgSO_4_, 0.05 mM 2-mercaptoethanol) and 20% ONGP buffer (13.28 mM ONGP, 82 mM Na_2_HPO_4_, 18 mM NaH_2_PO_4_). Absorbance was determined at 410 nm by the FLUOstar optima microplate reader. 

### 4.8. Flow Cytometry

To study myelin phagocytosis, isolated myelin (10 mg/mL) was fluorescently labelled with 12.5 μg/mL 1,1″-diotadecyl-3,3,3′,3′,-tetramethylindocarbocyanide perchlorate (DiI; Sigma-Aldrich) for 30 min at 37 °C, as previously described [43]. MDMs were pretreated with GW9662 (25 µM) or DMSO for 30 min, followed by an incubation with DiI-labeled myelin (100 µg/mL, 1.5 h) at 37 °C, 5% CO_2_. DiI fluorescence was measured using a FACS Calibur flow cytometer (BD biosciences, Erembodegem, Belgium).

### 4.9. Cholesteryl Ester (CE) Determination

MDMs were preincubated with GW9662 (25 µM) or DMSO for 30 min, followed by an incubation with myelin (100 µg/mL) for 24 h. CEs were measured using the Amplex^TM^ Red Cholesterol Assay Kit (Thermo Fisher Scientific), according to manufacturer’s instructions. Briefly, cells were washed twice with PBS after treatment and lysed in Amplex^TM^ Red reaction buffer. Amplex^TM^ Red working solution, with or without cholesterol esterase was added to the cell lysate and incubated at 37 °C protected from light. After 30 min, fluorescence was measured at an excitation wavelength of 540 nm and an emission wavelength of 590 nm using the FLUOstar optima microplate reader. CE concentration was calculated by subtracting free cholesterol concentration from total cholesterol concentration. 

### 4.10. Oil Red O (ORO) Staining and Quantification

MDMs were preincubated with GW9662 (25 µM) or DMSO for 30 min, followed by an incubation with myelin (100 µg/mL) for 24 h. Cells were fixed in 4% paraformaldehyde (Sigma-Aldrich) for 20 min at RT. Neutral lipids were stained using ORO (Sigma-Aldrich) in 60% isopropanol for 10 min. Following incubation, unbound particles were washed away with deionized water. For the ORO staining, coverslips were counterstained with Mayer’s hematoxylin for 60 s to visualize nuclei. Afterwards, cells were rinsed in running tab water and mounted on microscope slides with aqueous mounting solution. Representative images were taken using a Leica DM2000 LED microscope (Leica Microsystems, Wetzlar, Germany). For ORO quantification, ORO was extracted by incubating the cells with isopropanol for 10 min, while shaking. Absorbance was measured using a microplate reader (BIORAD iMark^TM^ Microplate Reader) at 510 nm.

### 4.11. Statistical Analysis 

MS patients were diagnosed based on the 2017 McDonald criteria [44]. Multiple linear regression models for each dependent response variable were fitted using SPSS Statistics 25.0 (SPSS, Chicago, IL, USA). A backward-elimination approach was performed in each multiple linear regression model until all remaining variables had *p* < 0.05. Data were statistically analyzed using GraphPad Prism v6 (GraphPad Software, La Jolla, CA, USA) and are reported as mean ± standard error of the mean (S.E.M.). D’Agostino-Pearson omnibus normality test was used to test normal distribution. One-way analysis of variance (ANOVA) with Tukey’s multiple comparison correction, two-way ANOVA with Sidak’s multiple comparison correction, or two-tailed unpaired Student’s *t*-test were used for normally distributed data sets. The Mann–Whitney analysis or one-way ANOVA with Dunn’s multiple comparison correction was used for data sets which did not pass normality. * *p* ≤ 0.05, ** *p* ≤ 0.01, and *** *p* ≤ 0.001.

## Supplementary Materials

The following supplementary materials are available online at https://www.mdpi.com/1422-0067/21/23/9329/s1, Figure S1: Negative correlation for LXRβ and age, Figure S2: Nuclear receptor expression in monocytes of SP-MS patients and in vitro differentiated monocyte-derived macrophages, Table S1: Descriptive statistics for the study population, Table S2: List of primer sequences used for RT-qPCR.

## Abbreviations

ABCA1	ATP-binding cassette transporter A1
ABCG1	ATP-binding cassette transporter G1
ANOVA	Analysis of variance
CD36	Cluster of differentiation 36
CE	Cholesteryl ester
CNS	Central nervous system
CPT1	Carnitine palmitoyl transferase 1
CSF	Cerebrospinal fluid
DiI	1,1″-diotadecyl-3,3,3′,3′,-tetramethylindocarbocyanide perchlorate
DMSO	Dimethylsulfoxide
EAE	Experimental autoimmune encephalomyelitis
ELISA	Enzyme-linked immunosorbent assay
FA	Fatty acids
HC	Healthy control
LD	Lipid droplet
LXR	Liver X receptor
MDM	Monocyte-derived macrophage
MS	Multiple sclerosis
NCoR	Nuclear receptor co-repressor
NR	Nuclear receptor
ORO	Oil red O
PBMC	Peripheral blood mononuclear cell
PHA	Phytohemagglutinin
PLIN2	Perilipin 2
PPAR	Peroxisome proliferator-activated receptor
RR-MS	Relapsing-remitting MS
RT-qPCR	Real-time quantitative PCR
RXR	Retinoid X receptor
SCD1	Stearoyl-CoA desaturase-1
S.E.M.	Standard error of the mean
SP-MS	Secondary progressive MS
SR-A1	Scavenger receptor-A1
SR-B1	Scavenger receptor-B1
