# Supplementary material for: Altered PPARγ Expression Promotes Myelin-Induced Foam Cell Formation in Macrophages in Multiple Sclerosis
Source: Int J Mol Sci. 2020 Dec 7;21(23):9329. doi: 10.3390/ijms21239329 (PMC7731422; doi:10.3390/ijms21239329)
Supplement: Supplementary file 1 [file ijms-21-09329-s001.pdf]

Supplementary Materials

Altered PPARγ Expression Promotes Myelin-Induced Foam Cell Formation in Macrophages in Multiple Sclerosis

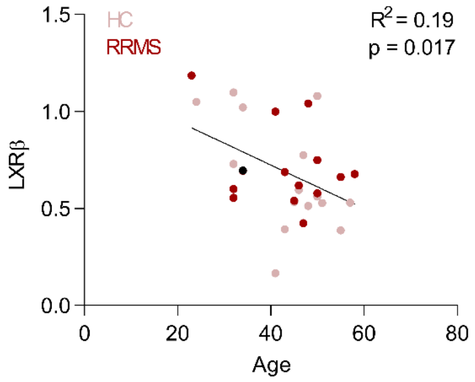

Figure S1. Negative correlation for *LXRβ* and age. The regression line describing the relationship between LXRβ and age in healthy controls and RR-MS patients (*n* = 30 total; *n* = 15 healthy controls and RR-MS patients) is shown.

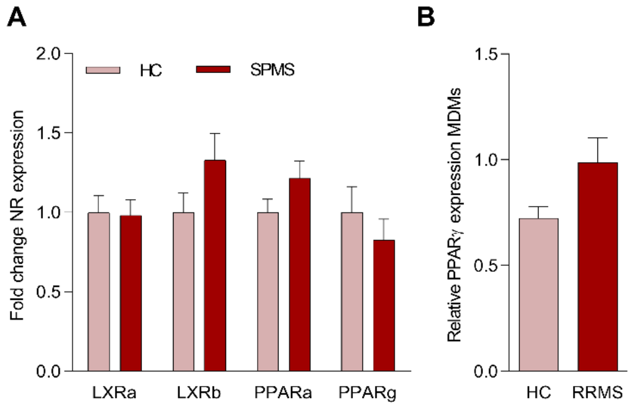

Figure S2. Nuclear receptor expression in monocytes of SP-MS patients and in vitro differentiated monocyte-derived macrophages. (**A**) Basal mRNA expression of *LXR*- and *PPAR*-isoforms in monocytes from age- and gender-matched healthy controls and secondary-progressive MS patients (SP-MS) (*n* = 9). (**B**) mRNA expression of *PPARγ* in macrophages differentiated from monocytes isolated from healthy controls and relapsing-remitting MS patients (RR-MS) (*n* = 11/group). Values represent the mean ± S.E.M. Statistical significance was assessed using one-way ANOVA with Dunn's multiple comparison correction (**A**) and unpaired student *T*-test (**B**). n represents the total number of donors included in the experiment.

Table S1. Descriptive statistics for the study population.

	HC vs. RR-MS		HC vs. SP-MS	
	**HC (*N* = 15)**	**RR-MS (*N* = 15)**	**HC (*N* = 7)**	**SP-MS (*N* = 7)**
Age [a] (years)	43.67 ± 9.4	43.67 ± 9.7	54.29 ± 7.34	54.43 ± 6.8
Male gender, (%)	3 (20%)	3 (20%)	3 (43%)	3 (43%)
EDSS [a]		2.77 ± 1.9		5.58 ± 1.69
Therapy (*n*)				
No treatment		3		2
Natalizumab		4		1
Interferon-beta		6		1
Cyclophosphamide		1		1
Glatiramer acetate		1		
Metotrexate				2

[a] Mean ± Standard Deviation. HC: healthy control, RR-MS: relapsing-remitting multiple sclerosis, SP-MS: secondary progressive multiple sclerosis, EDSS: expanded disability status scale.

Table S2. List of primer sequences used for RT-qPCR.

Gene	Forward Primer	Reverse Primer
h-ABCA1	AAC-GAG-ACT-AAC-GAG-GCA-ATC	ACA-CAA-TAC-CAG-CCC-AGA-AC
h-ABCG1	CCA-GAA-GTC-GGA-GGC-CAT-C	AAG-TCC-AGG-TAC-AGC-TTG-GCA
h-CD36	GCA-GCT-GCA-TCC-CAT-ATC-TA	GGG-TAC-GGA-ACC-AAA-CTC-AA
h-CypA	AGA-CTG-AGT-GGT-TGG-ATG-GC	TCG-AGT-TGT-CCA-CAG-TCA-GC
h-IL6	GAG-GAG-ACT-TGC-CTG-GTG-AA	GCT-CTG-GCT-TGT-TCC-TCA-CT
h-LXRα	CTT-GCT-CAT-TGC-TAT-CAG-CAT-CTT	ACA-TAT-GTG-TGC-TGC-AGC-CTC-T
h-LXRβ	CAC-GAG-ACA-GAG-TGT-ATC-ACC-TTC	AGA-TGT-TGA-TGG-CGA-TGA-GCA-G
h-PLIN2	TGT-GAG-ATG-GCA-GAG-AAC-GGT	CTG-CTC-ACG-AGC-TGC-ATC-ATC
h-PPARα	GTG-GCT-GCT-ATC-ATT-TGC-TGT-G	CAC-ATG-TAA-ATA-CCC-TCC-TGC-AT
h-PPARγ	GAT-GTC-TCA-TAA-TGC-CAT-CAG-GTT	GGA-TTC-AGC-TGG-TCG-ATA-TCA-CT
h-RPL13A	AAG-TTG-AAG-TAC-CTG-GCT-TTC-C	GCC-GTC-AAA-CAC-CTT-GAG-AC
h-RXRα	CCC-TGT-CAC-CAA-CAT-TTG-CC	AGA-AGT-GTG-GGA-TCC-GCT-TG
h-RXRβ	GAA-GCT-CAG-GCA-AAC-ACT-AC	TGC-AGT-CTT-TGT-TGT-CCC
h-SCD1	GTA-CCG-CTG-GCA-CAT-CAA-CTT	TTG-GAG-ACT-TTC-TTC-CGG-TCA-T
h-SRA1	TCC-TCG-TGT-TTG-CAG-TTC-TC	GCA-ATT-CTT-CGT-TTC-CCA-CT
h-TBP	TAT-AAT-CCC-AAG-CGG-TTT-GC	GCT-GGA-AAA-CCC-AAC-TTC-TG
h-TNFα	AGC-CCA-TGT-TGT-AGC-AAA-CC	TGA-GGT-ACA-GGC-CCT-CTG-AT
h-YWHAZ	CTT-GAC-ATT-GTG-GAC-ATC-GG	TAT-TTG-TGG-GAC-AGC-ATG-GA
h-ABCA1	AAC-GAG-ACT-AAC-GAG-GCA-ATC	ACA-CAA-TAC-CAG-CCC-AGA-AC
h-ABCG1	CCA-GAA-GTC-GGA-GGC-CAT-C	AAG-TCC-AGG-TAC-AGC-TTG-GCA
